# Ultrasound as a noninvasive tool for monitoring reproductive physiology in male Atlantic salmon (*Salmo salar*)

**DOI:** 10.14814/phy2.14167

**Published:** 2019-07-09

**Authors:** Ingun Næve, Maren Mommens, Augustine Arukwe, Jonni Virtanen, Md. Enamul Hoque, Elin Kjørsvik

**Affiliations:** ^1^ AquaGen AS Trondheim Norway; ^2^ Department of Biology NTNU Trondheim Norway; ^3^ Clewer Aquaculture Oy Turku Finland; ^4^ Department of Oceanography University of Chittagong Chattogram Bangladesh

**Keywords:** Animal welfare, histology, male reproduction, sex hormones, ultrasound

## Abstract

We examined the potential for ultrasound as a noninvasive tool for maturation monitoring in Atlantic salmon (*Salmo salar*) males. Ultrasound examination and measurements were compared to common practices for maturation monitoring such as gonadosomatic index (GSI), sex hormone analysis, and histological analysis of spermatogenesis. There were significant correlations (*R* ^2^ = 0.68, *P* < 0.01) between ultrasound‐based measurements of the left testis and total testes weight and GSI, and ultrasound could be used for noninvasive GSI measurements. Echogenicity of ultrasound images corresponded to the histological stages observed, which added nuance to ultrasound‐based GSI measurements during final weeks preceding stripping. We propose that ultrasound can be used as an alternative to more invasive methods for sexual maturation monitoring in wild and farmed Atlantic salmon males. Using ultrasound technology, we have established a quick and noninvasive method that could reduce the number of stressful handlings and unwanted sacrifice of broodfish required for maturation monitoring in Atlantic salmon males.

## Introduction

The salmonid species are of great cultural and economic value, and knowledge and control of their reproduction is an important step both in management of wild, endangered populations and for commercial production. In the wild, Atlantic salmon (*Salmo salar*) return from seawater migration to their natal river for spawning (Hansen et al., 1993). During upstream river migration, decreasing photoperiod and water temperature induces final maturation of eggs and sperm and initiates spawning (Heggberget, 1988; Webb and McLay, 1996). In broodfish production, light and temperature control is used to advance and delay maturation in order to secure year‐round production and supply of fertilised eggs for customer demands (Pankhurst and King, 2010; Taranger et al., 2010; Vikingstad et al., 2016). Gonadosomatic index (GSI) is an invasive method that is most commonly used to monitor sexual maturation in fish. It is based on gonad weight as a percentage of total body weight, and the method requires unwanted sacrifice of valuable broodfish. In farmed broodstock, it is therefore often performed on deceased fish, which might not be representative for the population.

The GSI method for monitoring male maturation (Schulz et al., 2006; Fjelldal et al., 2011) is used alone or in combination with other invasive and semiinvasive methods such as histological analysis (Dziewulska and Domagaƚa, 2003, 2005), gene expression in relevant tissues (Melo et al., 2014) and single or repeated blood sampling for plasma sex hormone analysis (Youngson et al., 1988; Sakai et al., 1989). Blood sampling has the advantage of being less invasive but involves time‐consuming and costly analysis which requires tagging of individual fish and repeated handlings. Crowding and netting involved in handlings can induce stress response (Waring et al., 1992; Carey and McCormick, 1998) and have negative effects on fish health (Pickering and Pottinger, 1989; Fast et al., 2008) and reproductive outcome (Campbell et al., 1992; Allyn et al., 2001). Insertion of an endoscope in the abdominal cavity for sexing and maturation monitoring gives instant results, but comes with increased risk of infection and internal bleedings (Ortenburger et al., 1996; Swenson et al., 2007), which also is the case for cutting several centimeter‐long incisions for visual inspection of gonads (Melillo Filho et al., 2016). In search of noninvasive methods for maturation monitoring, sex steroid assays from holding water (Ellis et al., 2013), scoring of external morphology (Youngson et al., 1988), and computed tomography (Müller et al., 2004) have been suggested. None of these methods appear to be precise or practical enough for maturation monitoring at present.

Ultrasound is a noninvasive method for imaging of internal organs and tissues based on reflection of emitted sound waves from organs back to the transducer (Kossoff, 2000), giving grey‐scale images that have been used for sexing and male maturation monitoring in several species such as rainbow trout (*Oncorhynchus mykiss*; Evans et al. (2004)), European eel (*Anguilla anguilla*; du Colombier et al., 2015) and Chinese sturgeon (*Acipenser sinensis*; Du et al., 2017). These methods range in complexity from sexing of immature males based on exclusion (Newman et al., 2008), to descriptions of testes appearance in ultrasound images at different stages of maturity (Colombo et al., 2007; Wildhaber et al., 2007) and measurements such as external ultrasound‐based testis length and area in cross section ultrasound images (Evans et al., 2004; Bryan et al., 2007). In male Atlantic salmon, ultrasound has been used for sexing by exclusion and there have been attempts at measurement of gonad diameter for determination of maturity status (Reimers et al., 1987; Mattson, 1991).

Inspired by our previous work regarding use of ultrasound for maturation monitoring in females (Næve et al., 2018), we compared ultrasound examinations of Atlantic salmon males with established methods for maturation monitoring such as plasma sex hormone and histological analyses. The goals of this study were to a) establish ultrasound as a noninvasive tool for maturation monitoring in Atlantic salmon males and b) examine if ultrasound can replace more invasive methods for maturation monitoring such as blood and tissue sampling.

## Materials and Methods

### Ethics statement

Fish included in this experiment were reared according to the Norwegian aquaculture legislation. Euthanasia was performed according to European directive 2010/63/EU, Annex IV. According to Norwegian and European legislation concerning animal research, experimental conditions and sedation in this study are practices undertaken for the purpose of recognized animal husbandry, and therefore formal approval of these experiments by the Norwegian Animal Research Authority (NARA) was not requested.

### Fish husbandry

Atlantic salmon fry of the AquaGen strain were first fed in February 2012 and transferred to seawater as 1‐year‐old smolts in March 2013. During the seawater phase, fish were fed according to appetite (Ewos Opal 110/112 and Ewos Opal Breed 3500 from September 2014). Fish were reared under natural photoperiod, except for two periods of continuous light during the first and second seawater winters to prevent grilsing and to advance maturation, respectively (described in Næve et al. (2018)). In May 2015, fish were transferred to indoor circular freshwater tanks (8‐m diameter, 1.5‐m depth, 60 m^3^) for final maturation and stripping. Autumn light and temperature signals were given from freshwater transfer. In August 2015, a temperature drop was given to induce final maturation. Water temperature (see also Næve et al. (2018)) and O_2_ levels were registered daily at 3‐ and 6‐m depth during the seawater phase (58 to 116 % and 75 to 148 %, respectively) and freshwater rearing (97 to 114 %).

### Experimental design

Fish samplings started from September 2014, and 10 to 20 males (Table S1) were sampled monthly until the temperature drop (see fish husbandry), when five males were sampled weekly until running milt was observed in all sampled males (02.09.15). Before sex could be identified by external traits, ultrasound was used to identify males (see ultrasound specifications, below). Fish were sacrificed with an overdose of tricaine methanesulfonate (200 mg/L, Pharmaq, Norway) before the spinal cord was severed. Using heparinised vacuum tubes (Terumo, Japan), blood samples were drawn from the caudal vein and centrifuged at 2.4 x 1000 g for 10 min at 4 °C (Micro Star 17R, VWR, USA). Plasma was collected and stored on ice for 1 to 5 h prior to freezing at −80°C. Body weight was registered to the nearest 20 g using a digital scale (SFE 60K20IMP, Kern & Sohn GmbH, Germany) and length to the nearest centimeter (cm) using a measurement tape. From January 2015, ultrasound length of left testis was measured by placing the ultrasound probe at the clavicle and a ruler at the basis of the pectoral fin and moving the probe in a posterior direction while tracing the left testis in the ultrasound image. Ultrasound‐based left testis length was registered as distance traveled along the ruler by the ultrasound probe. Cross‐section ultrasound images (one to four per individual) of left testis were captured from each individual. Testes were gently dissected out and length of left testis and testis weight (left and total) was registered to the nearest 0.1 g using a digital scale (MFD, A&D, Japan and Scanvaegt DS‐673SS, Denmark). Cross‐sectional tissue samples (0.5 to 1 cm thick) were cut from the anterior half of the testis and fixed in 4 % formaldehyde solution in phosphate buffer (pH 6.9, Merck Millipore, Germany).

### Ultrasound specifications

Ultrasound examination was performed using a MyLab Alpha ultrasound machine (Esaote, Italy) with a linear array probe (3 to 13 MHz) placed at the clavicle. Examinations were performed at 3 to 5 MHz, with focus at 25 to 40 mm and gain (signal amplification) of 80 to 90 %.

### Hormonal analysis

Plasma levels of 11‐keto testosterone (11‐KT), maturation inducing hormone (MIH) and testosterone (T) were measured using enzyme immuno‐assay (EIA) kits (Cayman chemical, USA). Steroid sex hormones were extracted from frozen plasma samples using diethyl ether. Briefly, 500‐µL plasma was thoroughly mixed with diethyl ether (1:4 plasma:solvent) by vortexing. The phases were separated by freezing in liquid nitrogen, and the organic phase was decanted. The process was repeated, and the combined organic phase was evaporated overnight at 25°C. The dry extracts containing steroid hormones were resuspended in 500 µL of EIA buffer and frozen at −80°C pending analysis. Samples were analysed in duplicate as described by kit manufacturer. Absorbances were read at 410 nm for all three hormones using a Cytation 5 Imaging plate reader (BioTek Instruments, USA). Plasma steroid levels were calculated from standard curves fitted with linear regression of logit transformed data as instructed by the manufacturer.

### Histology

Tissue for histological analysis was fixed and stored in phosphate buffered formaldehyde at 4°C until dehydration by a tissue processor (TP1020, Leica Biosystems Nussloch GmbH, Germany). Testis slices were embedded in paraffin and sectioned at 4‐µm thickness using a microtome (2055/RM2255, Leica biosystems, Nussloch GmbH, Germany), before hematoxylin and eosine (H&E) staining. Glass slides were scanned at 20x magnification with a digital slide scanner (NanoZoomer, Hamamatsu photonics, Japan) and images viewed and exported from scanner software (NDP, Hamamatsu photonics, Japan) as jpg.‐files. Ten images were exported from each slide, each covering an area of approximately 0.71 mm^2^. A track map in the scanner software was used to avoid overlapping images.

Spermatogenesis was evaluated in three randomly chosen images from individuals selected for further analysis. A point grid of 112 crosses covering 6500 µm was overlaid with each image using Grid plugin for ImageJ (National Institutes of Health, USA). Cells and tissues in testis sections were classified according to Dziewulska and Domagaƚa (2003, 2005), Leal et al. (2009), Schulz et al. (2010) and Melo et al. (2014). Using a cell counter plugin for ImageJ, number of points (intersection of each cross) hitting each tissue type was counted. Area fraction for each tissue or cell type was calculated as number of points hitting each tissue type divided by total number of points.

Spermatogenic maturity index (SMI) was introduced by Tomkiewicz et al. (2011) as a method for evaluating the spermatogenic progress in histology slides of testis. SMI ranges from 0 (only testicular somatic cells present) to 1 (only spermatozoa present) and was calculated by weighting area fractions (F) of each tissue or cell type, according to the following equation:

$$\text{SMI}=0.0{\text{F}}_{\text{Ts}}+0.25{\text{F}}_{\text{Sg}}+0.5{\text{F}}_{\text{Sc}}+0.75{\text{F}}_{\text{St}}+1.0{\text{F}}_{\text{Sz}}$$

Sc, spermatocytes; Sg, spermatogonia; St, spermatids; Sz, spermatozoa; Ts, testicular somatic cells.

Further classification of substages within each spermatogenic stage was not performed as it was not possible with the applied method.

### Data analysis and statistics

Data were prepared using Microsoft Excel (Microsoft, USA). Figures were made using SigmaPlot 14 (Systat Software, USA). GSI, gonad weight as percent of body weight, was calculated as.

$$\text{GSI}=\left(\text{gonad weight}/\text{body weight}\right)\times 100$$

Condition factor, K, was calculated as.

$$\text{K}=100000\times \left(\text{weight}/\left({\text{length}}^{3}\right)\right)$$

At September throughout November 2014 samplings, total testes weights were not registered. There was a linear relationship between left and total testes weight at the rest of the samplings (Pearson’s correlation, *R* ^2^ = 0.992, *P* < 0.01). The following equation was used to calculate total testes weight for September throughout November 2014 samplings, and estimated total testes weight was used for GSI calculation for these samplings.

$$\text{Total testes weight}=\left(1.80\times \text{left testis weight}\right)+1.20$$

Variation in maturation (observed as GSI) was low during the first months (September 2014 throughout February 2015) of this study. Therefore, the number of males analysed for sex hormones and testis development represented by histological sections was reduced for these samplings (Table S1). From September 2014 throughout February 2015 samplings, five and six individuals were selected randomly for histology and sex hormone analysis, respectively. From March until June 2015, the five males with the highest and lowest GSI were analysed for sex hormones and histology. Statistical analyses were performed using IBM SPSS Statistics 25 (IBM, USA). Data were tested for normality using Shapiro–Wilk test, with *P* < 0.05 indicating nonnormal data. GSI, SMI, and sex hormone data were nonnormal and included some outliers, they were tested for differences between groups using Kruskal–Wallis H‐test followed by Dunn’s test with Bonferroni correction for pairwise comparisons (*P* < 0.05 for SMI and GSI and *P* < 0.01 for 11‐KT, MIH, and T). Linear and exponential ultrasound‐based GSI models were fitted using regression curve estimation, with *P* < 0.05. As these data were nonnormal, included outliers and did not have symmetrical distributions, the Sign test (*P* < 0.05) with Bonferroni correction was used to identify differences between the estimated GSI based on ultrasound measurement and the GSI measured by conventional method for each sampling. Correlations between SMI, sex hormone plasma levels, GSI, and ultrasound‐based GSI models were performed using Pearson product moment correlation, *P* < 0.05.

Data were rearranged into four groups according to GSI (<1; 1 to 2.9; 3 to 4.9 and 5 to 7) rather than monthly samplings to study differences in levels of SMI and sex hormones between these groups. Data were not normally distributed (Shapiro–Wilk test, *P* < 0.05), and differences between GSI groups were tested using Kruskal–Wallis H‐test followed by Dunn’s test with Bonferroni correction for pairwise comparisons (*P* < 0.05 for SMI and MIH, *P* < 0.01 for 11‐KT and T).

## Results

**Figure 1:**
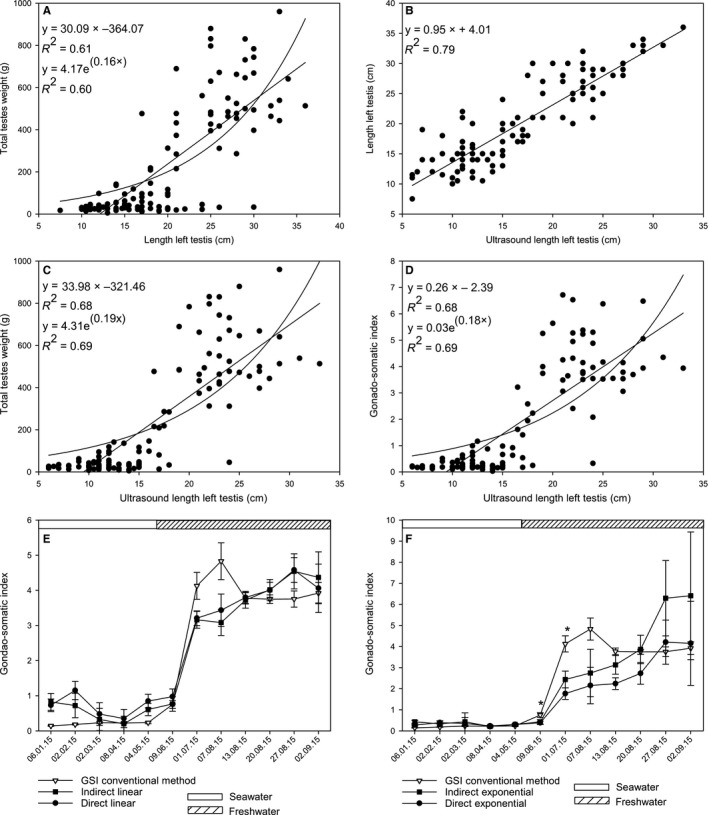
Ultrasound‐based estimation of gonadosomatic index in Atlantic salmon males during the last year before stripping. Correlation (Pearson’s correlation) between testis length and weight (A) and ultrasound length of testis (B). Correlations between ultrasound length of testis and total testis weight (C) and gonadosomatic index (GSI; D) were used to make direct (calculating GSI directly from ultrasound measurement) and indirect (calculating GSI by estimating ovary weight first) linear and exponential models for GSI estimation. GSI estimated using direct and indirect linear (E) and exponential models (F). Horizontal bars at the top (E and F) represent rearing conditions and data in E and F are mean ± SEM. Asterix in F represent significant differences between indirect exponential model and GSI in June, and between both exponential models and GSI in July (Sign test with Bonferroni correction, *P* < 0.05).

There was a strong correlation between length of left testis and total testis weight (Figure 1A, Pearson’s correlation; *R* ^2^ = 0.61, *P* < 0.01), and between length of left testis (Figure S1) and ultrasound measured length of left testis (Figure 1B, *R* ^2^ = 0.79, *P* < 0.01). Ultrasound measured left testis length correlated strongly with total testis weight (Figure 1C, *R* ^2^ = 0.68, *P* < 0.01) and GSI (Figure 1D, *R* ^2^ = 0.68, *P* < 0.01) and linear and exponential models for estimating total gonad weight and GSI using ultrasound‐based length of left testis were fitted. Applying these models, GSI was estimated indirectly using total testis weight calculated from ultrasound measurement and registered body weight, or directly from ultrasound measurement of left testis (Figure 1C and 1, respectively). Both linear models followed each other closely, and overestimated GSI (nonsignificant) during the seawater phase, while they first under‐ and then overestimated GSI during the freshwater phase (Figure 1E). The exponential models followed GSI calculated by conventional method closely during the seawater phase. Indirect exponential model significantly under‐estimated GSI in June and July, and then greatly overestimated (nonsignificant) GSI prior to stripping (Figure 1F). Direct exponential model significantly underestimated GSI for the July sampling, but estimated GSI more accurately in the final weeks before stripping.

**Figure 2:**
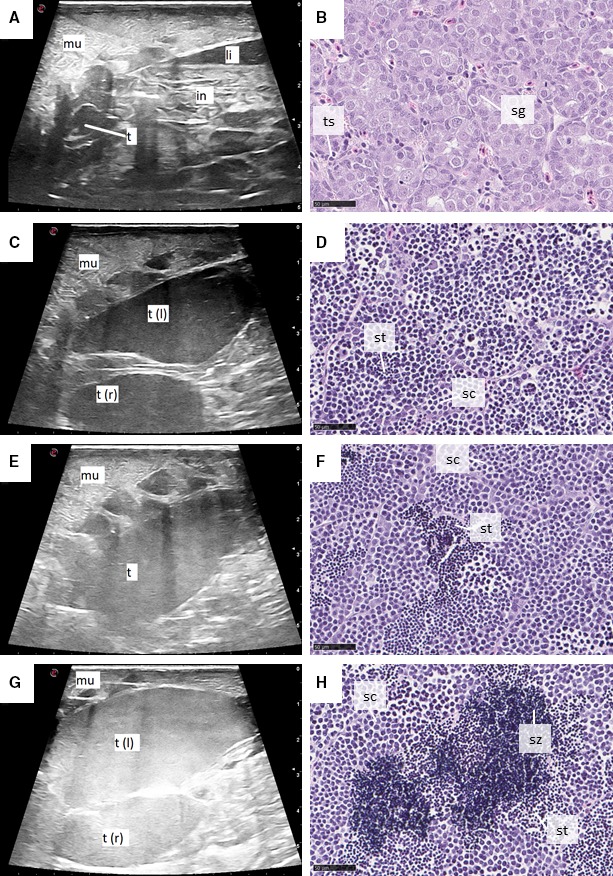
Spermatogenesis in Atlantic salmon males during the last year before stripping, represented by ultrasound images and histological sections. Ultrasound‐based left testis length was measured from January 2015, when testes were small in ultrasound images (A) and testis histology was dominated by spermatocytes and testicular somatic cells. Growth in testis size accompanied by presence of spermatocytes and spermatids (C and D) was observed in June 2015. In July 2015, change in echogenicity gave grey shade of testis in ultrasound images (E) and spermatids were observed in some histological sections (F). From July, males where testes were bright grey, almost white in ultrasound images were observed (G), these males often had spermatozoa present in histological sections (H). Scalebar (right) in ultrasound images is 5 cm. Scalebar in histology images is 50 µL. in, intestine; li, liver; mu, muscle; sc, spermatocyte; sg, spermatogonia; st, spermatid; sz, spermatozoa; t, testis; t (l), left testis; t (r), right testis; ts, testicular somatic cells.

**Figure 3:**
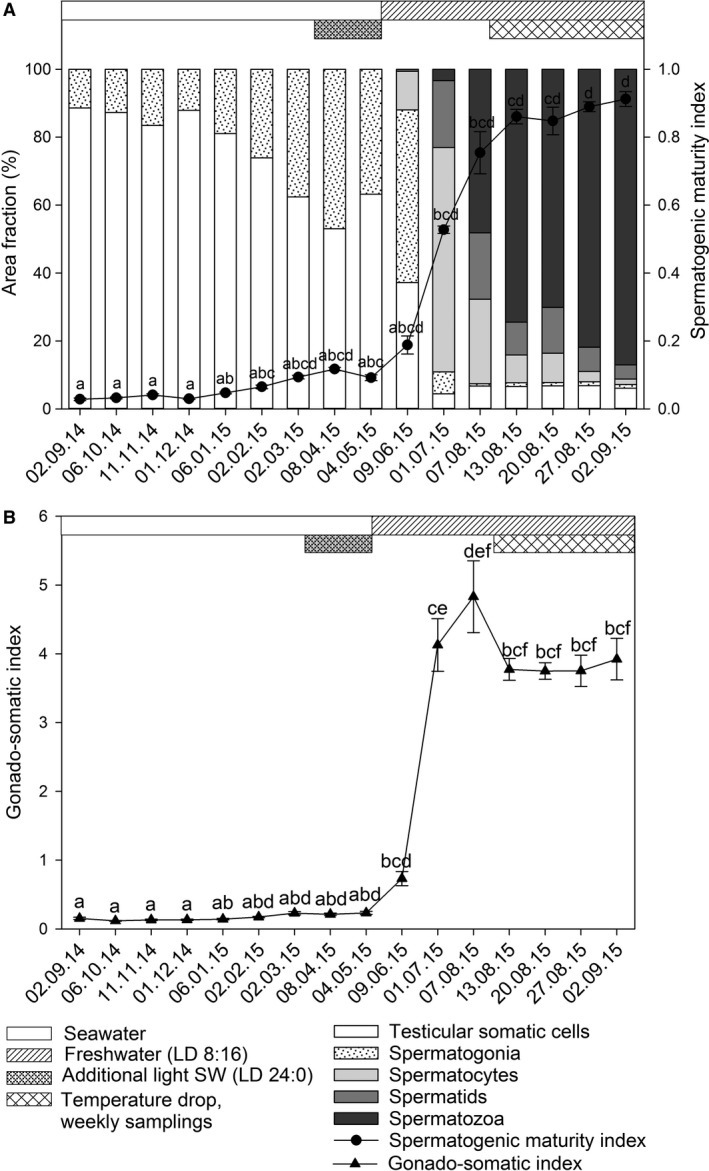
Area fractions of stages in spermatogenesis and spermatogenic maturity index (SMI) and gonadosomatic index (GSI) in Atlantic salmon males during the last year before stripping. SMI (A) and GSI (B) data are mean ± SEM. Bars in A represent fractions of cells and tissues in different stages of testis maturation. Letters indicate significant differences (Kruskal–Wallis H test) between samplings, *P* < 0.05 for SMI and GSI. Horizontal bars indicate rearing conditions. SW, seawater.

Left testis could not be discerned from internal organs and adipose tissue in ultrasound images during September throughout November 2014 samplings. During these months, sexing was done by exclusion as ovaries were visible in ultrasound images. From December 2014, left testis was observed in some individuals as a black, oval, or circular structure near the intestine and kidney. From January 2015, it was possible to measure ultrasound‐based left testis length in some individuals (Figure 2A). During seawater phase, little growth in left testis size was observed in ultrasound images. In histological sections from this period (Figure 2B), area fraction of spermatogonia and testicular somatic cells increased (11 to 37%) and decreased (89 to 63%), respectively (Figure 3A). At the first sampling after freshwater transfer (June 2015), left testis was black in ultrasound images, with increased size (Figure 2C) and spermatocytes were observed in histological sections from some individuals (Figures 2D, 3A). In the July 2015 sampling, testes in several males had outgrown the footprint of the ultrasound probe, and we were not able to capture the whole left testis in the cross‐section ultrasound image. At the July sampling, only one male had testis that appeared black in the ultrasound image, and left testis of most males appeared grey (Figure 2E). In these males, spermatocytes and spermatids could be observed in histological sections (66 and 20%, respectively, Figure 3A), while area fractions of spermatozoa were very low (1 to 3%, Figures 2F, 3A). In July, two individuals had testes that were bright grey, almost white in ultrasound images (Figure 2G), and these individuals had spermatozoa that accounted for ≥10% of testicular area fraction (Figures 2H, 3A). During August and September 2015 an increasing number of males with bright grey or white testis were observed. During the same period, the area fraction of spermatozoa increased to complete dominance (87 %) at stripping (Figure 3A).

During the seawater phase the mean GSI of Atlantic salmon males was stable at low levels (Figure 3B). At the first sampling after freshwater transfer in June 2015, GSI had increased slightly, followed by an exponential increase to a peak value of 5 in early August 2015 (Figure 3B). GSI declined to July 2015 levels and was stable at approximately 4 during the rest of the freshwater phase, including at final maturation. Mean SMI was low during seawater phase but showed a slight increasing trend during the final months in seawater (Figure 3A). After freshwater transfer SMI increased exponentially from 0.2 in June to 0.9 at stripping in September 2015. GSI was strongly correlated (Pearson’s correlation) to SMI and plasma levels of T and 11‐KT (Figure S2) while the correlation to MIH was moderate (Table 1). Both linear models for ultrasound‐estimated GSI had strong correlation to SMI, T, and 11‐KT and moderate correlation to MIH. Exponential ultrasound GSI models had strong correlation to SMI and moderate correlation to plasma levels of T, 11‐KT, and MIH (Table 1).

**Table 1:** Correlations between GSI, SMI, and plasma levels of the sex hormones T, 11‐KT, and MIH in male Atlantic salmon during the last year before final maturation.

	GSI conventional method	Indirect linear US‐GSI	Indirect exponential US‐GSI	Direct linear US‐GSI	Direct exponential US‐GSI
SMI	0.86	0.85	0.65	0.84	0.64
T	0.68	0.62	0.41	0.62	0.41
11‐KT	0.57	0.55	0.35	0.54	0.34
MIH	0.38	0.45	0.44	0.41	0.41

Numbers are R, *P* < 0.01 for all correlations (Pearson’s correlation). 11‐KT, 11‐keto testosterone; GSI, gonadosomatic index; MIH, maturation inducing hormone; SMI, spermatogenic maturity index; T, testosterone; US‐GSI, ultrasound‐based gonadosomatic index. Full correlation matrix can be found in Table S2.

**Figure 4:**
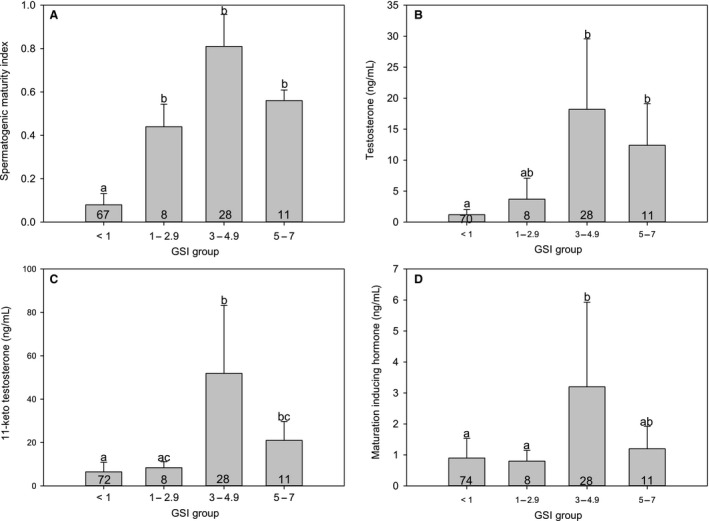
Levels of spermatogenic maturity index (SMI; A) and testosterone (T; B), 11‐keto testosterone (11‐KT; C) and maturation inducing hormone (MIH; D) in Atlantic salmon males grouped according to gonadosomatic index (GSI). Data are mean ± SD. Numbers at the base of each bar represents n. Letters indicate significant differences between GSI groups (Kruskal–Wallis H‐test), *P* < 0.05 for MIH and SMI, and *P* < 0.01 for 11‐KT and T. GSI group < 1 includes individuals from September 2014 throughout June 2015 samplings, GSI group 1 – 2.9 includes individuals from June and July 2015 sampling, GSI group 3 – 4.9 includes individuals from July, August and September 2015 samplings, and GSI group 5 to 7 includes individuals from July and 07.08.2015 samplings.

Rearranging males in groups according to GSI showed similar trends in SMI and all three sex hormones measured. SMI (Figure 4A) and plasma 11‐KT, T,and MIH (Figure 4B–D) levels were low in males with GSI less than 1 and increased to a peak in males with GSI ranging 3 to 4.9. Males with the largest gonads, namely those with GSI between 5 and 7, had lower levels of sex hormones and SMI than males with GSI between 3 and 4.9.

## Discussion

Using ultrasound technology, we have established a quick and noninvasive method that could reduce number of stressful handlings and unwanted sacrifice of broodfish required for maturation monitoring in Atlantic salmon males.

The ultrasound‐based GSI method requires one measurement of left testis length, and calculations can easily be performed in an excel sheet, giving instant results. Indirect and direct ultrasound‐based GSI methods that were established here both have linear and exponential alternatives available. The indirect approach calculates GSI based on measured body weight and total testis weight estimated from ultrasound‐based left testis length measurement, while the direct approach estimates GSI from ultrasound measurement of left testis length. Linear indirect and direct methods for ultrasound‐based GSI are suitable for GSI estimation after freshwater transfer although it should be kept in mind that they deviate somewhat from GSI measured by conventional method. Direct and indirect exponential models underestimate GSI too much during early freshwater phase (June and July). Thus, these models are not recommended for maturation monitoring. For GSI estimation during seawater phase, exponential models are recommended, as these follow GSI calculated by conventional method closely in seawater. However, none of the models presented here were able to represent the peak and decline in GSI observed during the weeks preceding stripping.

In early reports on use of ultrasound in salmonids, simple sexing of maturing individuals was achieved (Martin et al., 1983; Reimers et al., 1987). Subsequently, attempts at maturation assessment using area measurements in cross‐section ultrasound images were attempted. However, these methods involved some uncertainties (Mattson, 1991; Evans et al., 2004). In our experience, performing such measurements using ultrasound equipment can be cumbersome and time‐consuming, prolonging handling time and stress for each fish. Using ultrasound‐based GSI method reported here, results are obtained within seconds, reducing handling time and stress to the broodstock. To ease handling of large male broodfish and keep handling stress and subsequent risk of reduced fish health and welfare at a minimum (Fast et al., 2008; Tort, 2011; Conte, 2004), we strongly suggest that ultrasound‐based GSI measurements should be performed using anesthesia (Iversen et al., 2003).

A corresponding method for GSI estimation was recently established for Atlantic salmon females (Næve et al., 2018). In the present study, *R* ^2^‐values for ultrasound‐based GSI model in males were lower than those reported in salmon females. In addition, the observed variations were higher than those observed in females, giving a less accurate GSI estimate in males. Ultrasound‐based ovary length measurements are quite easy to perform because ovaries are clearly defined and increase in size in an anterior to posterior direction as maturation progresses. Atlantic salmon testes are elongated structures spanning the length of the abdominal cavity from early life stages (Laird et al., 1978). Therefore, testes do not grow in length during sexual maturation as in females, but rather in thickness, starting anteriorly and narrowing toward the anus. When measuring left testis length, the thickened part was registered, and it could be challenging to define the end of testis thickening, both when using ultrasound and measuring tape. With practice and experience, accuracy of ultrasound‐based length measurement could probably be increased, giving more exact models and ultrasound‐based GSI measurements for male Atlantic salmon.

Echogenicity describes the amount of sound waves that are reflected to the ultrasound transducer from different tissues (Lieu, 2010). Low echogenicity gives a dark appearance of an organ or tissue in ultrasound images, while high echogenicity gives a bright grey or white appearance. During these samplings, we observed a change in testes echogenicity that was related to testis maturation. Not all males at the spermatozoa stage had bright grey or white testes in ultrasound images, but all males that had bright grey or white testes had spermatozoa present in histological sections. These differences in echogenicity could be used as a noninvasive method for maturation monitoring after freshwater transfer in Atlantic salmon males. At present, this method should be regarded as a semi‐objective approach for evaluating testes development using ultrasound. However, when coupled with computer‐assisted grey‐scale analysis techniques, it may be possible to quantify these observed differences in grey‐scale as has been done in human studies (Reimers et al., 1993; Pillen et al., 2007).

The high correlation observed between ultrasound‐based GSI and SMI and plasma sex hormone levels indicates that ultrasound is a promising noninvasive tool for identifying individual progression during spermatogenesis and high plasma androgen levels. Levels of sex hormones reported here were in accordance with previous findings in salmonid species (Fitzpatrick et al., 1986; Scott and Sumpter, 1989; Tveiten et al., 1998), although MIH concentrations were in the lower range of what has been previously reported (reviewed in Scott et al. (2010)). A decrease in GSI calculated by the conventional method was observed before final maturation, as has been observed in several other species such as brown trout (*Salmo trutta*; Billard, 1983) and rainbow trout (Scott and Sumpter, 1989; Prat et al., 1996). The decline in GSI during final maturation could be due to loss of cellular material during meiotic cleavage and maturation of spermatids into spermatozoa (Stanley, 1969; Schulz et al., 2010). Further, a germ cell loss of up to 30 % has been reported in Nile tilapia (*Oreochromis niloticus*) during spermiogenesis (Vilela et al., 2003; Schulz et al., 2005).

Given that GSI peaks and declines during the month before running milt was observed, using only GSI or ultrasound‐based GSI during this period might not be sufficient to conclude on reproductive stage alone. For example, a male with GSI of around four could either be at the spermatocyte and spermatid stage with GSI still increasing and quite some time until final maturation (typically seen in July), or he could be at the spermatozoa stage, approaching spermiation and be ready for stripping within short time (typically end of August). In the absence of additional information, GSI or ultrasound‐based GSI is not informative enough to certainly determine maturation status at this point. Adding observations of echogenicity gives more nuance. Using both ultrasound‐based GSI value and observed echogenicity (i.e., black/ grey/ bright white testis in ultrasound image), ultrasound examination can provide a more accurate estimate of the progression of maturation in individual male Atlantic salmon.

In conclusion, this study has shown that ultrasound is suitable for maturation monitoring in male Atlantic salmon. The ultrasound‐based GSI models provide valuable information about progress in maturation, levels of sex hormones and spermatogenic development. In addition, it is specific enough for grading purposes when supplemented with observation of echogenicity. The method is quick and easy to perform for trained personnel, and it requires only basic knowledge of fish anatomy of the operator. Using ultrasound technology, more invasive methods are not needed, therefore we believe the use of ultrasound will reduce stress and improve welfare during maturation monitoring of both farmed and wild fish, including endangered salmonids.

## Conflict of Interest

All authors declare no conflict of interest.

## Supporting information




**Figure S1**. Atlantic salmon left testis lenth measurement. During sexual maturation testes thickness increase in an anterior to posterior direction. The clearly thickened part of testis was measured when measuring testis length.

**Figure S2**. Plasma levels of testosterone (A), 11‐keto testosterone (B) and maturation inducing hormone (C) in Atlantic salmon males during the last year before stripping. Data are mean ± SEM. Horizontal bars at the top indicate rearing conditions. Letters indicate significant differences (Kruskal‐Wallis H‐test) between samplings, *P *< 0.01. SW, seawater.

**Table S1**. Body weight (mean ± S.E.M.) and K factor (mean ± S.E.M.) for Atlantic salmon males sampled during the last year before final maturation and stripping.
**Table S2**. Full correlation matrix between GSI, SMI and plasma levels of the sex hormones T, 11‐KT and MIH in male Atlantic salmon during the last year before final maturation.
