# Supplementary figures and images for: Ultrasound as a noninvasive tool for monitoring reproductive physiology in male Atlantic salmon (Salmo salar)
Source: Physiol Rep. 2019 Jul 9;7(13):e14167. doi: 10.14814/phy2.14167 (PMC6640606; doi:10.14814/phy2.14167)

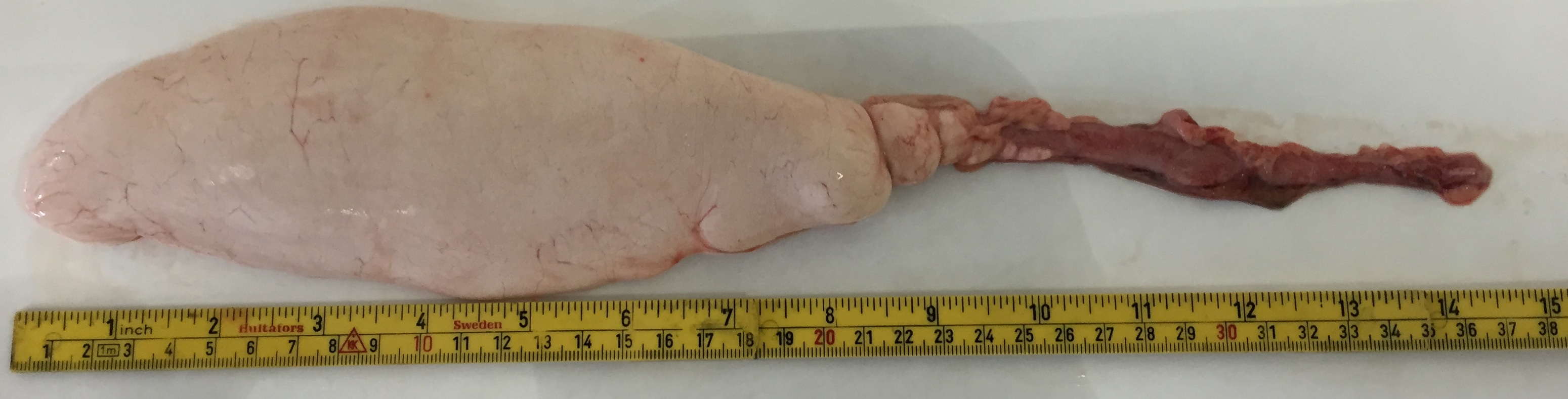

Supplement: Supplementary file 1 — Figure S1. Atlantic salmon left testis lenth measurement. During sexual maturation testes thickness increase in an anterior to posterior direction. The clearly thickened part of testis was measured when measuring testis length. [file PHY2-7-e14167-s001.tif]

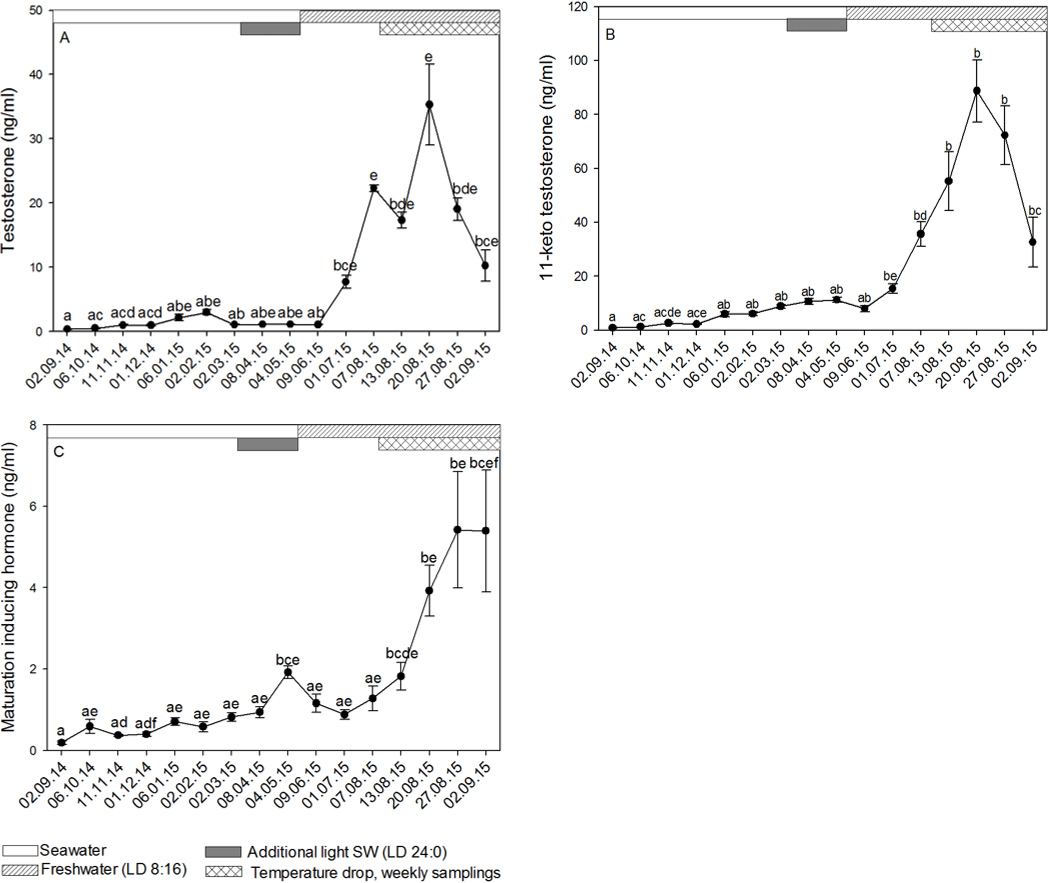

Supplement: Supplementary file 2 — Figure S2. Plasma levels of testosterone (A), 11‐keto testosterone (B) and maturation inducing hormone (C) in Atlantic salmon males during the last year before stripping. Data are mean ± SEM. Horizontal bars at the top indicate rearing conditions. Letters indicate significant differences (Kruskal‐Wallis H‐test) between samplings, P < 0.01. SW, seawater. [file PHY2-7-e14167-s002.tif]
