# Supplementary material for: Ultrasound as a noninvasive tool for monitoring reproductive physiology in male Atlantic salmon (Salmo salar)
Source: Physiol Rep. 2019 Jul 9;7(13):e14167. doi: 10.14814/phy2.14167 (PMC6640606; doi:10.14814/phy2.14167)
Supplement: Supplementary file 3 — Table S1. Body weight (mean ± S.E.M.) and K factor (mean ± S.E.M.) for Atlantic salmon males sampled during the last year before final maturation and stripping. Table S2. Full correlation matrix between GSI, SMI and plasma levels of the sex hormones T, 11‐KT and MIH in male Atlantic salmon during the last year before final maturation. [file PHY2-7-e14167-s003.docx]

Table S1. Body weight (mean ± S.E.M.) and K factor (mean ± S.E.M.) for Atlantic salmon males sampled during the last year before final maturation and stripping. Number of individuals analysed for sex hormones (testosterone, 11-keto testosterone and maturation inducing hormone) and histology is also listed. 11-KT, 11-keto testosterone; MIH, maturation inducing hormone; T, testosterone

| Date (n) | Body weight (kg) | K factor | Histology (n) | 11-KT (n) | MIH (n) | T (n) |
| --- | --- | --- | --- | --- | --- | --- |
| 02.09.14 (16) | 6.5 ± 0.2 | 1.18 ± 0.02 | 5 | 6 | 6 | 6 |
| 06.10.14 (19) | 7.2 ± 0.3 | 1.12 ± 0.02 | 5 | 6 | 6 | 6 |
| 11.11.14 (10) | 9.1 ± 0.5 | 1.23 ± 0.03 | 5 | 6 | 6 | 6 |
| 01.12.14 (12) | 9.5 ± 0.5 | 1.27 ± 0.04 | 5 | 6 | 6 | 6 |
| 06.01.15 (11) | 9.4 ± 0.5 | 1.18 ± 0.04 | 5 | 6 | 6 | 6 |
| 02.02.15 (14) | 12.6 ± 0.7 | 1.40 ± 0.03 | 5 | 6 | 6 | 6 |
| 02.03.15 (14) | 12.2 ± 0.9 | 1.30 ± 0.07 | 10 | 10 | 10 | 9 |
| 08.04.15 (12) | 15.3 ± 0.5 | 1.45 ± 0.03 | 10 | 10 | 10 | 9 |
| 04.05.15 (13) | 14.1 ± 0.7 | 1.31 ± 0.03 | 10 | 10 | 10 | 9 |
| 09.06.15 (20) | 13.5 ± 0.4 | 1.20 ± 0.02 | 10 | 9 | 10 | 9 |
| 01.07.15 (20) | 13.3 ± 0.5 | 1.14 ± 0.02 | 20 | 20 | 20 | 20 |
| 07.08.15 (5) | 14.2 ± 0.6 | 1.08 ± 0.02 | 5 | 5 | 5 | 5 |
| 13.08.15 (5) | 13.2 ± 0.5 | 1.09 ± 0.05 | 5 | 5 | 5 | 5 |
| 20.08.15 (5) | 12.9 ± 0.9 | 1.08 ± 0.04 | 5 | 5 | 5 | 5 |
| 27.08.15 (5) | 13.2 ± 0.6 | 1.06 ± 0.05 | 5 | 5 | 5 | 5 |
| 02.09.15 (5) | 12.2 ± 1.1 | 1.02 ± 0.03 | 5 | 5 | 5 | 5 |

Table S2: Full correlation matrix between GSI, SMI and plasma levels of the sex hormones T, 11-KT and MIH in male Atlantic salmon during the last year before final maturation. Numbers are R, p < 0.01 for all correlations (Pearson’s correlation). 11-KT, 11-keto testosterone; GSI, gonado-somatic index; MIH, maturation inducing hormone; SMI, spermatogenic maturity index; T, testosterone; US-GSI, ultrasound-based gonado-somatic index.

|  | Total gonad weight | GSI conventional method | Indirect linear GSI | Indirect exponential GSI | Direct linear GSI | Direct exponential GSI | T | 11-KT | MIH | SMI |
| --- | --- | --- | --- | --- | --- | --- | --- | --- | --- | --- |
| Total gonad weight |  | 0.98 | 0.79 | 0.57 | 0.82 | 0.59 | 0.67 | 0.56 | 0.35 | 0.84 |
| GSI conventional method | 0.98 |  | 0.84 | 0.59 | 0.83 | 0.59 | 0.68 | 0.57 | 0.38 | 0.86 |
| Indirect linear US-GSI | 0.79 | 0.84 |  | 0.81 | 0.97 | 0.80 | 0.62 | 0.55 | 0.45 | 0.85 |
| Indirect exponential US-GSI | 0.57 | 0.59 | 0.81 |  | 0.80 | 0.99 | 0.41 | 0.35 | 0.44 | 0.65 |
| Direct linear US-GSI | 0.82 | 0.82 | 0.97 | 0.80 |  | 0.83 | 0.61 | 0.54 | 0.41 | 0.84 |
| Direct exponential US-GSI | 0.59 | 0.59 | 0.80 | 0.99 | 0.83 |  | 0.41 | 0.33 | 0.41 | 0.64 |
| T | 0.67 | 0.68 | 0.62 | 0.41 | 0.62 | 0.41 |  | 0.80 | 0.48 | 0.77 |
| 11-KT | 0.56 | 0.57 | 0.55 | 0.35 | 0.54 | 0.33 | 0.80 |  | 0.61 | 0.77 |
| MIH | 0.35 | 0.38 | 0.45 | 0.44 | 0.41 | 0.41 | 0.48 | 0.61 |  | 0.59 |
| SMI | 0.84 | 0.86 | 0.85 | 0.65 | 0.84 | 0.64 | 0.77 | 0.77 | 0.59 |  |
